# Forming social impressions from voices in native and foreign languages

**DOI:** 10.1038/s41598-018-36518-6

**Published:** 2019-01-23

**Authors:** Cristina Baus, Phil McAleer, Katherine Marcoux, Pascal Belin, Albert Costa

**Affiliations:** 1 Center for Brain and Cognition (CBC), Universitat Pompeu Fabra, Barcelona, Spain; 2 School of Psychology, University of Glasgow, Glasgow, UK; 3 Institut des Neurosciences de la Timone, UMR 7289, CNRS and Université Aix-Marseille, Marseille, France; 4 Institució Catalana de Recerca i Estudis Avançats (ICREA), Catalonia, Spain

## Abstract

We form very rapid personality impressions about speakers on hearing a single word. This implies that the acoustical properties of the voice (e.g., pitch) are very powerful cues when forming social impressions. Here, we aimed to explore how personality impressions for brief social utterances transfer across languages and whether acoustical properties play a similar role in driving personality impressions. Additionally, we examined whether evaluations are similar in the native and a foreign language of the listener. In two experiments we asked Spanish listeners to evaluate personality traits from different instances of the Spanish word “Hola” (Experiment 1) and the English word “Hello” (Experiment 2), native and foreign language respectively. The results revealed that listeners across languages form very similar personality impressions irrespective of whether the voices belong to the native or the foreign language of the listener. A social voice space was summarized by two main personality traits, one emphasizing valence (e.g., trust) and the other strength (e.g., dominance). Conversely, the acoustical properties that listeners pay attention to when judging other’s personality vary across languages. These results provide evidence that social voice perception contains certain elements invariant across cultures/languages, while others are modulated by the cultural/linguistic background of the listener.

## Introduction

When meeting people for the first time, we cannot avoid forming quick impressions about them, even when very limited information is available. We pay attention to how people look, dress and sound and use such information to form an impression about their personality. Faces play a significant role in this process, but other properties such as people’s voices also play a role^1,2^. Indeed, just listening to the way people say the word “Hello” is enough to evaluate various personality traits of the speaker, such as attractiveness, aggressiveness, and confidence^3^. These evaluations serve to build a social voice space that helps form rapid impressions and consequently triggers approach or avoidance behaviors. Such social voice space can be summarized by two main personality traits, one emphasizing trust/likeability/warmth and the second emphasizing strength/dominance^4^. These two traits seem to be very relevant when evaluating personality first impressions, as they have been replicated for faces and voices and do not seem to depend on other sources of information, such as the speech content provided by the speaker^5,6^. Despite the social voice space being subjectively similar to dimensional personality spaces for faces and group dynamics^4,6^ as well as to non-social utterances in voices^1,5^, how the space transfers for brief social statements across languages is unknown. This means that we do not fully understand how it applies when hearing your language and when hearing a foreign language.

The fact that the social voice space can be built just by listening to one word reveals that vocal properties of the speaker play a major role in the perception of personality from voices^3,7,8^. Acoustic-phonetic properties of individual voices are tightly intertwined with personality impressions and therefore represent a natural cue to social perception. As an instance, variations in pitch (akin to F0, F0 variation) are very powerful cues for listener’s judgments^9,10^. Low-pitch voices are judged as more dominant and attractive than high-pitch ones, especially for males^3,5,11–13^. As suggested by Ohala^14^, the relation between dominance and pitch stems from an innate specified “frequency code”, which associates high acoustic frequency with subordinate, nonthreatening traits and low acoustic frequency with dominant, aggressive or threatening traits. In Spanish, pitch has been related to credibility in radio advertisement, with a preference for females, which normally have a higher pitch than males^15^. Less clear is however the relationship between pitch and other personality traits. For instance, some studies have related trustworthiness with low-pitch voices, especially in the context of voting behavior^12^, while others have suggested the opposite or even no relationship^3,16,17^. Other acoustics more related to the roughness of the voice (harmonics-to-noise ratio, HNR an index of harmonic to irregular vocal components or “voice quality”) have been related to positive traits such as attractiveness or benevolence^18^, but the evidence is limited. The role of other voice properties (e.g., jitter/shimmer) on social evaluation remains still unclear. Thus, while it is clear that listeners pay attention to changes in speech rate or voice quality to form impressions, our understanding of which acoustics play a significant role in driving such impressions is somewhat limited. One of the most relevant aspects of the present study is the comparison of the same personality traits and acoustics across languages to determine how voice acoustics drive evaluation of personality traits. As such, the present article explores two issues: a) whether the two-dimensional social voice space is generalizable across languages and if so b) whether listeners of one language have a similar social voice space when listening to foreign language speakers. To explore said issues, we asked Spanish listeners to evaluate personality traits from Spanish voices (Experiment 1) and Scottish voices (see^3^; Experiment 2).

Concerning the issue of how generalizable the social voice space is, the question is not only whether the same dimensions are relevant across languages (e.g., dominance, valence/trustworthiness, etc.) but also whether people pay attention to the same acoustic features when building such a space. Using Scottish voices, McAleer *et al*.^3^ showed a high inter-rater consistency across listeners in their evaluations of perceived personality. Additionally, the Principal Component Analysis (PCA) revealed that the ratings of the eight personality traits clustered into two main components: likeability/trustworthiness (PC1 – named valence in the work of^6^) and dominance (PC2). If the organization of the traits observed in McAleer *et al*.^3^ corresponds to vocal cues universally exploited by speakers, perhaps as a consequence of evolutionary pressures^19^, then judgments of listeners when rating voices, irrespective of language, would be organized in a similar fashion^20^. This will be in line with the idea that the Big-Five personality dimensions are used in every known language to distinguish people (e.g,^21^). Within this framework, culture might also exert influence by accentuating or reducing some dimensions over others. For instance, Scherer^1^ revealed that listeners judging the personality of American and German voices identified sociability as the primary trait defining American voices while dominance was the most salient trait of German voices (see also,^22^). These results were interpreted as reflecting that relevant values for interpersonal relationships differ across societies: historically a sociable behavior was valued in American society while dominance-submission was valued in German society^23,24^. Similarly, Aaker, Benet-Martínez and Garolera^25^ revealed both similarities and differences between American and Spanish listeners when evaluating personality through commercial brands: while Spanish listeners valued passion from commercial brands, American listeners valued competence (see^26^, for a review).

Thus, in the present study, we first tested if the voice social space obtained in English, with valence and dominance as the main personality traits, extends to Spanish. Any difference across languages might well respond to social stereotypes specific to each culture modulating the relevance of traits and the underlying vocal characteristics when composing the social voice space. To explore this issue, we asked native Spanish listeners to rate different personality traits, by listening to the word “Hola (*hello* in English)” uttered by Spanish speakers.

As mentioned above, even with similar ‘voice space’ dimensions, judgments of what is trust or dominant per se may not be based on the same acoustic properties across languages/cultures. Considering that the relation between specific vocal properties and personality traits is culturally-influenced, this is a reasonable hypothesis. After all, differences between the prosodic patterns across languages may lead people to focus on different aspects of the acoustic signal when assessing personality traits.

For example, Peng *et al*.^2^, observed that Korean and American listeners used some acoustic properties (vocal speed) differently when evaluating speaker’s competence, while other properties such as loudness were treated similarly (see also^27^, for differences between Japanese and Dutch women in the use of pitch). Similarly, Babel & McGuire^28^ showed that despite attractivenes judgments highly correlated across listeners of different English dialects (California, western Canada), the phonetic features used to make those judgments differed across populations, suggesting that vocal expressions (at least for attractiveness) involve community-specific preferences. Other acoustics, however, seem to be less influenceable by the listener’s culture. Take pitch for instance. Its influence in dominance and attractiveness evaluations of males has been replicated across several languages/cultures^29^. In our attempt to determine the “universality” of the vocal social space, we should consider the influence of culture on how social perception is organized.

A secondary question related to the generalizability of the social voice space is whether listeners from a different language of the speaker similarly evaluate personality traits to that of native listeners. That is when hearing the same speakers, do listeners, irrespective of language, utilize the same personality traits and acoustics when forming a first impression? The limited research concerning this questions suggests that when assessing people’s personality, we tend to evaluate speakers of our native language as more intelligent, kind and as having a stronger personality than speakers of a foreign language^30^. Given the reduced familiarity with a foreign language and the potential particularities of their first language social voice space, it is an open issue whether these listeners extract the same personality traits when exposed to a foreign language. Although somehow indirect, evidence on emotion recognition suggests that the ability to recognize vocal emotions is governed both by language-independent (universal) and linguistic/cultural factors^31,32^. That is, listeners can infer the speaker’s emotional state (e.g., fear, anger) irrespective of the language, native or foreign, but there is an in-group advantage in recognizing vocally expressed emotions in the native language. If the same applies to speaker’s evaluation of personality traits, we should expect a similar two-dimensional social space for voices, for native and foreign listeners, but some differences in the weight of traits or the underlying vocal characteristics. To explore this issue, in Experiment 2 we asked native Spanish speakers to rate different personality traits, by listening to the word “Hello” uttered by Scottish speakers. These same utterances had already been rated by native Scottish listeners^3^, so we can compare whether the resulting social voice spaces of native and non-native listeners are similar.

In summary, in two experiments, we evaluated: (1) the generalizability of the social voice space across languages by testing Spanish listeners judging the Spanish word “Hola” and (2) the impact of foreign language on the creation of the social voice space, by testing Spanish listeners judging the English word “Hello”. In both experiments, we explored the personality dimensions driving the social voice space and the relation with the vocal properties of the voice.

## Experiment 1: Spanish listeners rating Spanish speakers

Following McAleer *et al*.’s study^3^, we asked native Spanish listeners to rate different instances of the word “Hola” uttered by 64 native Spanish speakers, according to different traits. Speakers and listeners were different individuals, and participants rated only one trait (e.g., aggressiveness, dominance, trust, etc.). If the social voice space obtained in English has some cross-linguistic stability, then we expect to find high inter-rater agreement among Spanish listeners. Additionally, we would expect that the PCA leads to the detection of two components and that the weights of the different traits in these components are similar to those observed in English.

### Methods

#### Participants

Native Spanish speaker students at the Universitat Pompeu Fabra rated the recorded materials (N = 279, mean age: 20.2 ± 2.5, 92 males). All of the participants were selected from the participants’ database of the Neurociences laboratory, which includes explicit consent to participate in the experiments. No other consent form was obtained from the participants. All methods were in accordance with the guidelines of the ethics committee at the University Pompeu Fabra and approval of the experimental protocol was obtained from the University ethical committee (CEIC-*Comité Étic d’Investigació Clínica*).

#### Materials

Sixty-four native Spanish speakers (22.1 ± 4.2 years, 32 males) from the Universitat Pompeu Fabra were recorded saying the word “Hola”. These speakers were born and raised in Spain and hence had peninsular accents. The extracted stimuli were normalized for power and loudness using PRAAT 6.0.14 and lasted an average of 319 ms ± 67.1 ms for male voices and 338 ms ± 60.0 ms for female voices, but such difference was not significant (p = 0.24). For subsequent acoustical analysis, we followed McAleer *et al*.,^3^ procedure and eight acoustic measurements were extracted from each token (considering its total duration) using PRAAT 6.0.14:

1. Mean fundamental frequency (F0) – a measure related to pitch, the periodic oscillation at the fundamental frequency of the vocal folds (Latinus & Belin, 2011).
2. Intonation, calculated by subtracting F0-min from F0-max, which indicates the pitch raise between the vowels of the word.
3. Glide, calculated by subtracting F0-start from F0-end.
4. Formant dispersion, which is the ratio between consecutive formant means, from F1 to F4, using the Burg linear predictive coding algorithm in PRAAT^33^. Maximum formant frequency was set to 5.5 kHz, window length = 0.025.
5. Harmonic-to-noise ratio (HNR), which indicates roughness in the voice, via the forward cross-correlation method (mean value; time step = 0.01 s; min pitch = 75 Hz; periods per window = 4.5).
6. Jitter, which measures local F0 variations^34^, via Average Relative Perturbation (RAP) measuring the average absolute difference between a period and the average of that period and its two neighbors’ periods (shortest period = 0.0001 s; longest period = 0.02 s; max. period factor = 1.3).
7. Shimmer^35^, a measure of amplitude variation, via the Amplitude Perturbation Quotient (APQ3) measuring the average absolute difference between a period’s amplitude and its neighbor’s average of, divided by the average (shortest period = 0.0001 s; longest period = 0.02 s; max. period factor = 1.3; max. amplitude factor = 1.6).
8. Alpha ratio, which measures the slope of the source spectral^3^ using the ratio of mean energy within low (0–1 kHz) vs. high frequencies (1–5 kHz) computed for the long-term average spectrum.

The selected acoustic measures can be divided in acoustics related to speech rate (F0, intonation, glide, dispersion) and acoustics related to voice quality (HNR, alpha ratio, jitter, and shimmer), thus covering a wide range of voice properties. However, this does not preclude that other unexplored acoustics might as well guide inferences about others’ personality traits. Importantly, there are studies showing correlations between the selected acoustic measures and impressions of a speaker’s personality (e.g., intonation-pleasantness^36^). Even more relevant, it allows us to directly compare the existence of voice acoustics as indicators of speaker’s personality across languages.

#### Procedure

Participants were asked to rate the 64 voices on the given personality trait. The personality traits were: aggressiveness, attractiveness, competence, confidence, dominance, likeability, trustworthiness, and warmth. Participants were assigned to one of the eight personality ratings pseudorandomly. First, the trustworthiness trait was completed. That is the first group of participants all rated trustworthiness. Trustworthiness is a trait that comes out in all social space analyses (faces, voices, bodies, groups) and therefore the most likely to be comparable across participants. For the rest of the traits, participants were pseudorandomly assigned to one of the remaining traits.

On each trial, participants heard through the headphones one of the voices and were given the following instructions: “Evalúa cuán {TRAIT} te parece esta persona, según su voz” (*Evaluate how {TRAIT} you think this person is according to/based on their voice*). A 9-point Likert scale, ranging from 1, extremely not {TRAIT} to 9, extremely {TRAIT} was used for the ratings. Each personality trait was rated by an average of 34.8 participants (range 30–41).

The presentation of male and female voices was blocked, and the order of the blocks counterbalanced across participants. During each gender block, each voice was presented twice in a randomized order. The experiment was performed using EPrime v2.0 and lasted approximately 10 to 15 minutes.

#### Exclusion Criteria

Participants born or raised outside of Spain (n = 4) were excluded. Additionally, on completion of the study participants were asked if they recognized any of the voices, and if so, they were excluded from the analyses (n = 16). Moreover, we used the two exclusion criteria from McAleer *et al’s*. article,^3^: (1) Participants with low internal consistency were excluded (n = 25, those that rate the same stimuli very differently—with more than a 2 point difference for more than two thirds of the voices) and (2) Participants that show little variability were excluded—those that give the same rating for more than 75% of the voices (n = 1). Thus, the final sample consisted of 233 participants (75 males) with an average of 29 participants per personality trait (range 24–34). The data was also evaluated without the exclusion criteria applied. The results were the same as those reported with the exclusion criteria applied.

#### Data Analysis

Following the original study and given the substantial differences in vocal acoustics between male and female voices, we analyzed the two sets of voices separately. The z-score for each participant was calculated for each gender separately and then submitted to a Principal Component Analyses (PCA) to explore the social voice space. Z-scores were used to assess inter-rater reliability using Cronbach’s Alpha.

### Results

All traits revealed good reliability across raters, with all alphas above 0.70 (see Table 1 for Chronbach’s Alpha of each trait).

**Table 1:** Cronbach alpha scores for Hola voices rated by Spanish listeners (collapsed by gender). N shown relates to sample size after exclusion criteria applied.

Social Trait	Cronbach Alpha	N
Aggressiveness	0.98	24
Attractiveness	0.95	26
Competence	0.96	32
Confidence	0.92	26
Dominance	0.77	31
Likeability	0.92	32
Trustworthiness	0.96	28
Warmth	0.94	34
Average	0.93	29.1

#### Male Voices PCA

**Figure 1:**
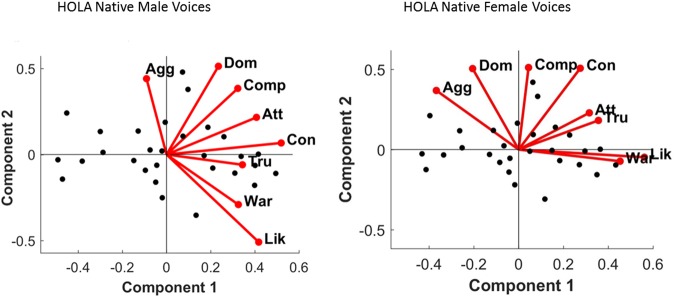
Principal Component Analysis solutions and main correlates of the Social Voice Space for “Hola” voices. (A) The two-dimensional solution of the PCA for male (left) and female (right) voices (black dots). Labels equate to: Agg – Aggressiveness; Att-Attractiveness; Comp- Competence; Con – Confidence; Dom – Dominance; Lik- Likeability; Tru – Trustworthiness; War – Warmth.

A two-dimensional solution explaining 89.6% of the variance was obtained for male voices. Principal component 1 (PC1) explained 66.3% of the variance and PC2, 23.2%. The two personality traits that most strongly loaded onto PC1 were confidence, (r = 0.95, p < 0.001) and trustworthiness (r = 0.94, p < 0.001), while aggressiveness (r = 0.79, p < 0.001) and dominance (r = 0.77, p < 0.001) were the main traits for PC2 (see Fig. 1 and Table 2). For PC1, all positive judgments (e.g., attractiveness, trustworthiness) had positive loadings, and all negative judgments (e.g., aggressiveness) had negative loadings, which has been extensively considered as an index of valence evaluation^6^.

**Table 2:** Loadings on the first two principal components of all social traits for male and female “Hola” voice PCAs, including variance explained.

Social Trait	Male PCA		Female PCA	
	Component 1	Component 2	Component 1	Component 2
Aggressiveness	−0.28	**0.79**	−0.77	0.58
Attractiveness	0.87	0.27	0.72	0.39
Competence	0.76	0.54	0.11	**0.91**
Confidence	**0.95**	0.07	0.55	0.76
Dominance	0.60	**0.77**	−0.47	**0.86**
Likeability	0.79	−0.57	**0.97**	−0.06
Trustworthiness	**0.94**	−0.10	**0.90**	0.34
Warmth	0.86	−0.45	**0.98**	−0.12
Variance Explained (%)	66.3	23.2	57.3	32.3

A second PCA was performed to obtain a solution unbiased concerning the main judgments composing the social voice space. To do so, those traits with the highest loadings were removed one at a time (aggressiveness, dominance, etc.). Pearson’s correlation coefficients are reported between the ratings of the excluded traits and the new PCA values. A good solution results from a high correlation to one component while no correlation to the other component. Confidence and trustworthiness ratings (all r’s > 0.9, p < 0.001) correlated with the new PC1 (of all ratings excluding confidence and trustworthiness respectively), and they did not correlate with the new PC2 (all r’s < 0.10, p = 0.5). Excluding aggressiveness, only the new PC2 correlated with aggressiveness (with PC1, r = −0.24, p = 0.18; with PC2, r = −0.67, p < 0.001). Conversely, excluding dominance, correlated with both the new PC1 and the new PC2 (with PC1, r = 0.51, p = 0.002; with PC2, r = −0.79, p < 0.001).

#### Female Voices PCA

A two-dimensional solution explaining 89.6% of the variance was obtained for female voices. Principal component 1 (PC1) explained 57.3% of the variance and PC2, 32.3%. The personality traits that most strongly loaded onto PC1 were warmth (r = 0.98, p < 0.001), likeability (r = 0.97, p < 0.001) and trustworthiness (r = 0.90, p < 0.001), while competence (r = 0.91, p < 0.001) and dominance (r = 0.86, p < 0.001) were the main traits for PC2 (see Fig. 1 and Table 2). As it was the case for male voices, for PC1 of female voices, all positive judgments (e.g., attractiveness, trustworthiness) had positive loadings, and all negative judgements (e.g., aggressiveness) had negative loadings, suggesting that valence summarized the first dimension.

The new PCA (excluding traits with the highest loadings) revealed that warmth and likeability highly correlated with the new PC1 (excluding warmth and likeability respectively; all rs >0.90; p < 0.001) while they did not correlate with the new PC2 (all rs <−0.19, p > 0.05). Excluding competence highly correlated with the new PC2 but not with PC1, while excluding dominance correlated with both the new PC1 and PC2 (competence with PC1, r = 0.07, p = 0.6; competence with PC2, r = 0.84, p < 0.001; dominance with PC1, r = −0.33, p = 0.05; dominance with PC2, r = 0.89, p < 0.001).

Given that our participant’s pool was skewed towards females (68% female participants), we evaluated the influence of the participants’ gender on the PCAs. To do so, we conducted new PCAs considering evaluations from male and female participants (see Supplementary Material). The results showed no influence of the gender of the listener on personality evaluation. Regardless of participants’ gender, valence and dominance were the main traits driving personality impressions from the voice.

#### Acoustic variation of Spanish voices and personality traits

We obtained the equivalent of McAleer *et al*.^3^, in Spanish regarding the two dimensions summarizing perceived personality from voices. The question now is whether the same acoustic measures are relevant in judging personality traits for Scottish and Spanish voices. To summarize McAleer’s *et al*. results, PC1 of male voices was mainly described by F0 and HNR. PC1 of female voices was influenced by HNR, intonation, and glide. For male voices, PC2 was primarily described by a combination of alpha, F0, HNR, and dispersion. For female voices, dispersion and F0 better characterized PC2. That is, measures related to pitch (F0, intonation) were related to trustworthiness and dominance, especially for male voices. High-pitch voices were considered more trustworthy both for male and female voices. In contrast, dominance was related to low-pitch for male voices and high-pitch for female voices. Following the original study, to evaluate the influence of acoustic measures in Spanish, stepwise regression analysis with the eight selected acoustic measures was conducted to explain the variance of the two-dimensional voice space. Unexpectedly, none of the analyses turned out to be significant. That is, none of the eight acoustic measures accounted for the variance explained in the PC1 and the PC2 dimensional solution obtained in Spanish, neither for male nor female voices.

**Figure 2:**
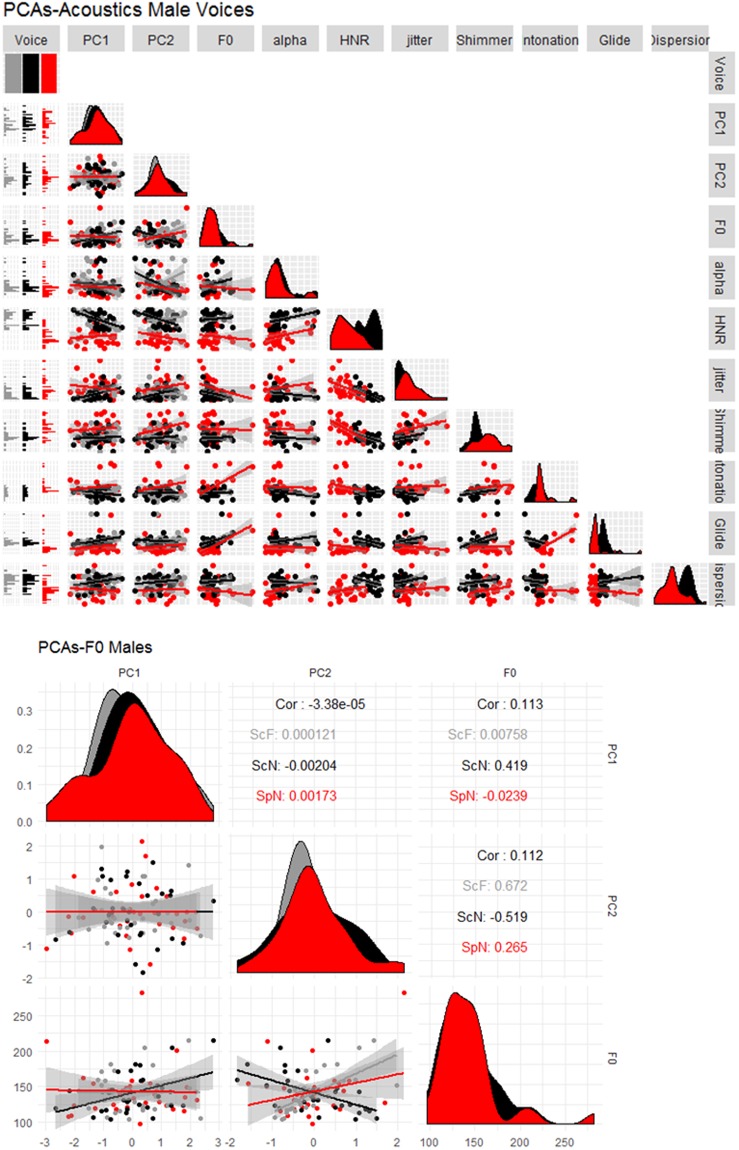
Upper panel. Correlation matrix and distribution density distribution (diagonal) of PC1 and PC2 of personality traits and voice acoustics for male voices. Black points represent Scottish voices in the original experiment (ScN: Scottish native), Gray points represent Scottish voices in Experiment 2 (ScF: Scottish foreign) and Red points represent Spanish voices in Experiment 1 (SpN; Spanish native). The lower panel is a zoomed in view of the correlation between PCs and F0 (Package GGally in R).

**Figure 3:**
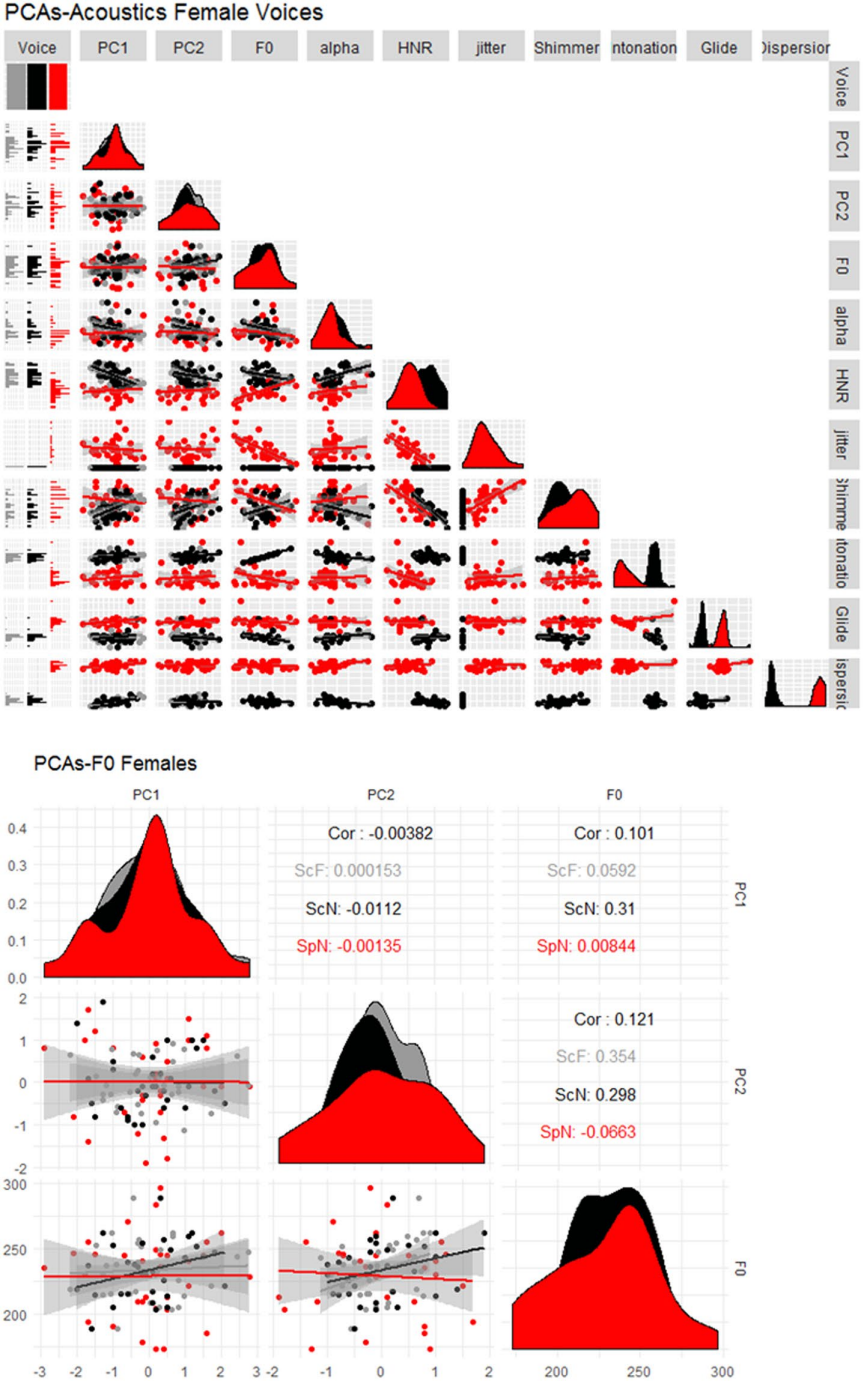
Upper panel. Correlation matrix and distribution density distribution (diagonal) of PC1 and PC2 of personality traits and voice acoustics for female voices. Black points represent Scottish voices in the original experiment (ScN: Scottish native), Gray points represent Scottish voices in Experiment 2 (ScF: Scottish foreign) and Red points represent Spanish voices in Experiment 1 (SpN; Spanish native). The lower panel is a zoomed in view of the correlation between PCs and F0 (Package GGally in R).

This result was surprising, especially considering the importance of pitch in personality evaluation for male voices^3,14^. We further investigated this null effect in several ways. First, we evaluated whether large variations in the acoustic measurements between Scottish and Spanish voices could explain the lack of effects obtained in Spanish. From the eight acoustic properties measured (F0, intonation, glide, dispersion, alpha, HNR, jitter, shimmer), Scottish and Spanish voices differed significantly for glide, dispersion, HNR, jitter, shimmer, and alpha (all with p < 0.04, except alpha p = 0.07). In contrast, F0 and intonation were similar between the two languages (all with p > 0.2). Thus, the fact that F0 values were similar between the two languages discard that cross-language differences are at the origin of the observed pattern of results (see Figs 2 and 3 for the distribution of the acoustics across languages for males and females respectively).

**Figure 4:**
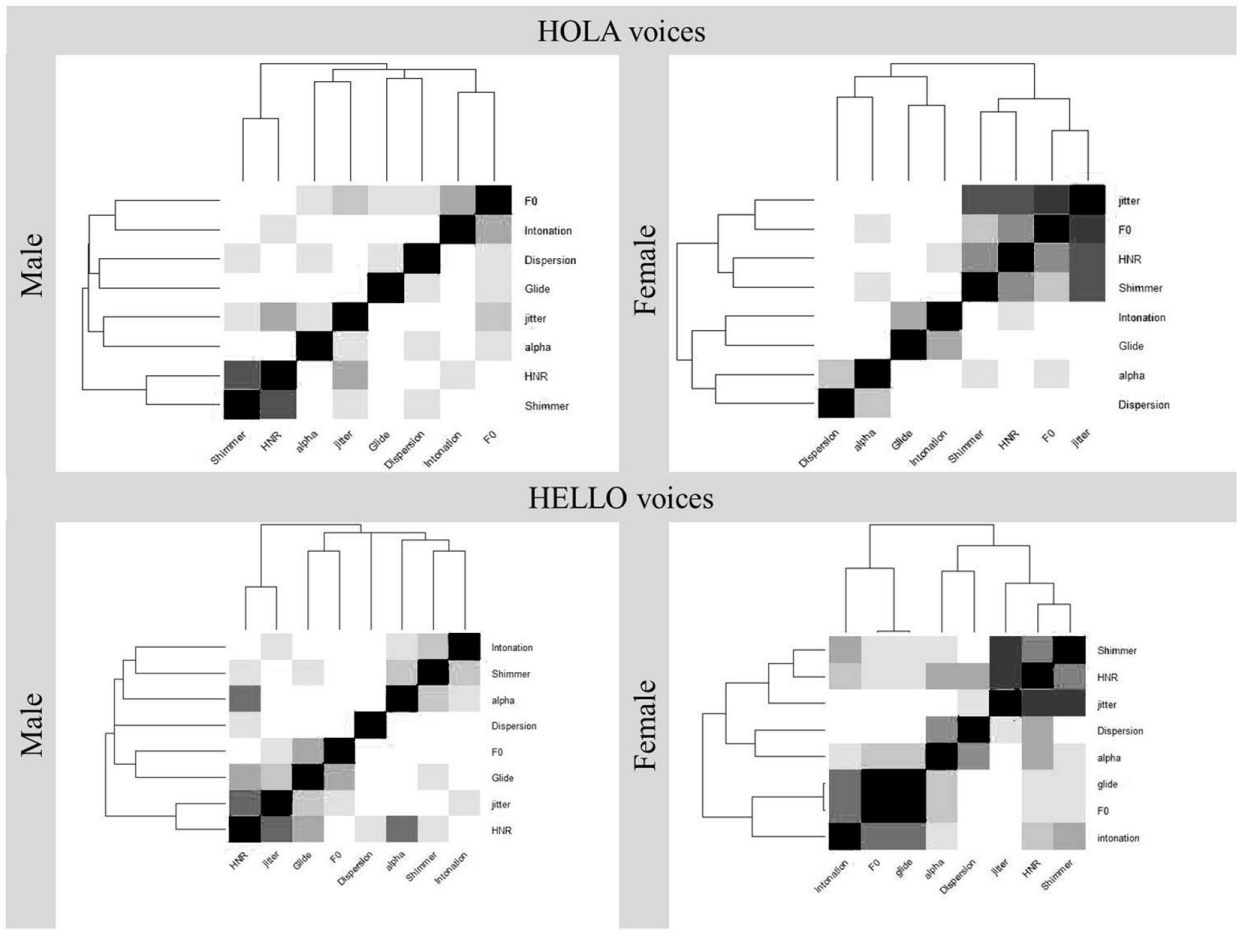
Spearman correlation matrix and hierarchical clustering of the voice acoustics in Experiment 1 and 2 (package ggplot2 in R). Black color represents one-to one correlations. The darker the grey, the stronger the correlation between acoustics. The order in which the acoustics are placed determines the ordered clusters.

Second, due to the large number of measures evaluated and their potentially high level of collinearity, we conducted a hierarchical clustering analysis to assess correlations between the acoustics employed (ggplot2 package in R). As can be seen in Fig. 4, acoustic measures are ordered and clustered according to Spearman correlations. For male voices, HNR and Shimmer and F0 and intonation formed the two main clusters. For female voices, the acoustics were grouped in three clusters: 1) jitter, F0, HNR and shimmer, 2) glide and intonation, and 3) dispersion and alpha. Since some of the acoustics were highly correlated, we tried to reduce collinearity conducting PCAs on the acoustics and then correlating the obtained PCAs with the ones obtained for personality traits. For male voices, HNR (r = 0.85, p < 0.001) and dispersion (r = 0.88, p < 0.001) loaded the most for PC1. For PC2, shimmer (r = 0.81, p < 0.001) and glide (r = −0.81, p < 0.001) were the acoustics that loaded the most. For female voices, HNR (r = 0.86, p < 0.001), shimmer (r = 0.84, p < 0.001) and intonation (r = −0.85, p < 0.001) loaded the most for PC1. For PC2, jitter was the acoustic that loaded the most (r = 0.73, p < 0.001). However, no correlation was obtained between PCAs of the acoustics and PCAs of the personality traits, neither for male nor female voices (all p’s > 0.14).

In sum, regardless of the analysis conducted, we did not obtain any evidence that the acoustic measures selected from McAleer *et al*., (2014) drove personality evaluations for Spanish voices.

### Discussion

The results of this study parallel those obtained in English by McAleer *et al*.^3^ regarding the evaluation of personality traits from voices. First, we found high inter-rater reliability for most of the traits, suggesting that people agree on personality impressions of an individual just by listening to the word “Hola” (see,^37,38^, for other studies using the word Hello). Second, the social voice space that results from the principal component analysis is similar to that observed in English, with a two-dimensional solution summarizing all the personality traits. The first dimension was comprised of trustworthiness and confidence for male voices, and trustworthiness, likeability, and warmth for female voices; both akin to a valence component^6^. The second dimension was mainly comprised of aggressiveness and dominance for male voices and competence and dominance for female voices.

These results support the notion that, as with faces, perceived personality in voices can be summarized by a two-dimensional space with two prominent traits, one emphasizing trust/likeability/warmth (i.e., valence or a positive/negative judgement) and a second trait emphasizing a strength, power and dominance judgement^3^ (see,^6,39,40^, for a similar interpretation for faces). Furthermore, our results show that the gender of the rater does not influence evaluations of the speaker’s voice. In contrast to previous reports^41,42^, ratings by both sexes were highly correlated, and the leading traits driving impressions were nearly the same for male and female listeners. Importantly, these results replicate those from McAleer *et al*.^3^, showing very similar results for male and female raters.

There were also subtle differences between Scottish and Spanish listeners in the traits that most loaded to each PCA. As shown in McAleer *et al*.^3^, across genders, PCA solutions differed mainly in the weighting of attractiveness. For male voices, attractiveness was more related to dominance (PC2) than to valence (PC1), while for female voices, attractiveness was uniquely related to valence. Subjective inspection of our original PCA (see Fig. 1) revealed that irrespective of gender, attractiveness in Spanish was related to valence (PC1) but not to dominance (PC2).

Remarkably, the most relevant difference between languages was reported on the acoustical properties that explained the social voice space. We found that none of McAleer *et al*.’s^3^ chosen vocal properties were related to personality ratings of Spanish voices. This suggests that native Spanish and Scottish listeners do not utilize the same acoustic measures when forming first impressions of novel speakers, despite subjective similarities in the social voice space that both use to make these decisions. Further discussion on this issue is raised in the General Discussion.

## Experiment 2: Spanish Listeners Rating Scottish speakers

In this experiment, we explored whether the reported two-dimensional social voice space for first impressions holds for listeners evaluating speakers of a foreign language.

In Experiment 2, Spanish listeners evaluated Scottish voices, therefore differences in the vocal properties of speech between the original experiment and ours do not hold. Interestingly, the question is whether the relationship between vocal properties and personality traits would be more English-like as in McAleer *et al*.’s^3^ experiment or more Spanish-like as in Experiment 1. That is, will Spanish listeners pay attention to the acoustic properties of the language of the speaker, regardless of it being their native or foreign language?

### Methods

#### Participants

A novel group of native Spanish speaker students at the Universitat Pompeu Fabra from the same pool as in Experiment 1 were used in this experiment (N = 305; 22.03 ± 4.5 years, 129 males). All had studied English for some years and claimed to be proficient in English. Participants were asked to evaluate their English proficiency on a scale from 1 to 7, with one indexing no knowledge of English and seven as native level (mean = 5.4, SD = 1.1). All of the participants were selected from the participants’ database of the Neurociences laboratory, which includes explicit consent to participate in the experiments. No other consent form was obtained from the participants. All methods were in accordance with the guidelines of the ethics committee at the University Pompeu Fabra (CEIP).

#### Materials

The same materials from McAleer *et al*.’s (2014) study were used, consisting of recordings from 64 English speakers (28.2 ± 10.2 years, 32 male) born and raised in Scotland, saying the word “Hello”. Recording settings were the same as in Experiment 1. For male voices, the stimuli lasted an average of 391 ms ± 65.1 ms, and for female voices, it lasted 390 ms ± 64.1 ms on average. Additionally, the same eight voice characteristics (1. mean F0, 2. intonation, 3. glide, 4. formant dispersion, 5. harmonic-to-noise, 6. jitter, 7. shimmer, and 8. alpha) were used in the analyses.

#### Procedure

The procedure was the same as in Experiment 1, with the only exception that the instructions were given in English.

#### Exclusion Criteria

The same exclusion criteria used in Experiment 1 were applied. Eight participants were excluded as they were not born or raised in Spain or declared that Spanish was not their preferred language. Besides, 39 participants were excluded from the analysis due to exclusion criteria 1 and 2 (37 participants according to exclusion criteria 1, and 2 participants according to exclusion criteria 2). Thus, the final pool of participants in Experiment 2 was 258 (106 males) with an average of 32 participants per personality trait (range 29–37).

#### Data Analysis

The same analyses as in Experiment 1 were performed.

### Results

The inter-rater reliability was quite high, with all alpha values above 0.90 (see Table 3 for Cronbach’s Alpha), indicating that attributions to voices of a foreign language appear very consistent across listeners and very similar to those reported for voices in the native language^3^.

**Table 3:** Cronbach alpha scores for Hello voices (McAleer *et al*., 2014) rated by Spanish listeners (collapsed by gender). N shown relates to sample size after exclusion criteria applied.

Social Trait	Cronbach Alpha	N
Aggressiveness	0.97	33
Attractiveness	0.97	30
Competence	0.96	37
Confidence	0.92	32
Dominance	0.93	32
Likeability	0.90	32
Trustworthiness	0.93	29
Warmth	0.97	33
Average	0.94	32.2

#### Male Voices PCA

**Figure 5:**
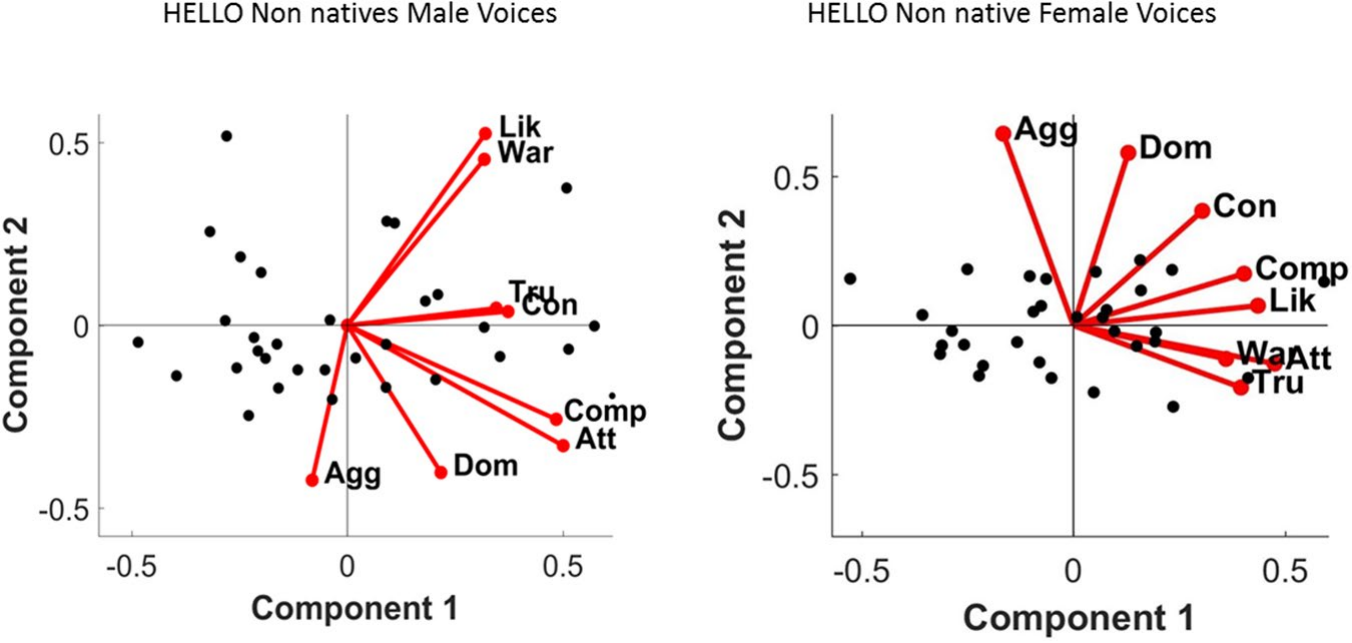
Principal Component Analysis solutions and main correlates of the Social Voice Space for “Hola” voices. (A) The two-dimensional solution of the PCA for male (left) and female (right) voices (black dots). Labels equate to: Agg – Aggressiveness; Att-Attractiveness; Comp- Competence; Con – Confidence; Dom – Dominance; Lik- Likeability; Tru – Trustworthiness; War – Warmth.

A two-dimensional solution explaining 90.4% of the variance was obtained for male voices. Principal Component 1 (PC1) explained 65.42% of the variance and PC2, 25.03%. The two personality traits that most strongly drove PC1 were competence (r = 0.93, p < 0.001) and trustworthiness (r = 0.93, p < 0.001), while aggressiveness (r = 0.86, p < 0.001) and dominance (r = 0.72, p < 0.001) were the main traits for PC2 (see Fig. 5 and Table 4).

**Table 4:** Loadings of the first two principal components for all social traits for male and female “Hello” voice PCAs, including variance explained.

Social Trait	Male PCA		Female PCA	
	Component 1	Component 2	Component 1	Component 2
Aggressiveness	−0.27	−**0.86**	−0.44	**0.86**
Attractiveness	**0.90**	−0.37	**0.94**	−0.13
Competence	**0.93**	−0.31	0.92	0.20
Confidence	0.89	0.06	0.73	0.46
Dominance	0.62	−**0.72**	0.38	**0.85**
Likeability	0.68	0.69	**0.93**	0.07
Trustworthiness	**0.93**	0.08	**0.93**	−0.24
Warmth	0.72	0.63	0.88	−0.14
Variance Explained (%)	65.4	25.03	69.1	17.3

As for Experiment 1, to establish summaries of the principal components, a second PCA was performed removing individual scales of those traits with the highest loadings within the two-dimensional space. The new analyses revealed that competence and trustworthiness highly correlated with PC1 (excluding competence and trustworthiness respectively; all rs > 0.85; p < 0.001), but only competence correlated with PC2 (competence: r = 0.44, p = 0.01; trustworthiness: r = 0.08, p = 0.6). Excluding aggressiveness, highly correlated with PC2 but not with PC1 (with PC1, r = −0.23, p = 0.1; with PC2, r = −0.80, p < 0.001), and dominance correlated both with PC1 and PC2 (with PC1, r = 0.55, p = 0.001; with PC2, r = 0.72, p < 0.001).

#### Female Voices PCA

A two-dimensional solution accounting for 86.46% of the variance was found for female voices—PC1 explained 69.15% of the variance and PC2 explained 17.31%. The personality traits that most strongly loaded in PC1 were attractiveness (r = 0.94, p < 0.001), trustworthiness (r = 0.93, p < 0.001) and likeability (r = 0.93, p < 0.001), while aggressiveness (r = 0.86) and dominance (r = 0.85) were the main traits in PC2 (Fig. 5). Correlations of the new PCAs revealed that attractiveness, trustworthiness and likeability highly correlated with PC1, when excluding the relevant trait (with PC1, all r’s > 0.88, p < 0.001; with PC2, all r’s < 0 0.07, p < 0.1). Both PC1 and PC2 correlated with aggressiveness and dominance when excluding the relevant trait (aggresiveness with PC1, r = −0.38, p = 0.03; with PC2, r = 0.78, p < 0.001; dominance with PC1, r = 0.33, p = 0.06; with PC2, r = 0.71, p < 0.001).

As for Experiment 1, our participant’s pool was skewed towards females (59% female participants). To evaluate the influence of the participants’ gender on the PCAs, we conducted new PCAs considering evaluations from male and female participants (see Supplementary Material). The results showed that the evaluation of personality traits does not seem to be influenced by the gender of the listener. Regardless of participants’ gender, valence and dominance seem to be the main traits driving personality impressions from voices.

#### Acoustic variation of Scottish voices and personality traits

As for Experiment 1, independently by gender of the voice, stepwise regression analyses were performed using eight acoustical measures to explain variance of PC1 and PC2. Regarding male voices, for PC1 a linear combination of HNR (b = −0.63, p < 0.001) and alpha (b = −0.28, p = 0.03) explained 60% of the variance (R = 0.79, F (2, 29) = 24.4, p < 0.001). Tests for multicollinearity indicated that a very low level of multicollinearity was present (*VIF* = 1.21 for alpha, *VIF* = 1.21 for HNR). For female voices, 22% of the variance of PC1 (R = 0.50, F (1, 30) = 10.1, p = 0.003) was explained by HNR (b = −0.20, p = 0.003). Regarding PC2, for male voices, 66% of the variance (R = 0.84, F (4, 27) = 16.1, p < 0.001) was accounted for by a combination of F0 (b = 0.71, p < 0.001, *VIF = *1.17), shimmer (b = −0.37, p = 0.002, *VIF = *1.09), alpha (b = 0.33, p = 0.006, *VIF = *1.09) and jitter (b = 0.31, p = 0.01, *VIF = *1.24). For female voices, shimmer (b = 0.56, p = 0.01) accounted for most of the variance of PC2 (29% variance; R = 0.56, F (1, 30) = 14.2, p = 0.001).

In sum, HNR influences the evaluation of personality traits related to valence (e.g., trustworthiness, warmth) for both female and male voices. Besides, dominance for male voices was associated with a higher pitch (F0) and with higher shimmer for female voices. [In Fig. 4, the correlation matrix of the acoustics for Scottish voices is provided. Given that some of the acoustics were highly correlated, we conducted PCAs of the acoustics and then correlated them with those of the personality traits. The new analysis confirmed the relation between acoustics and traits. PC1 of the acoustics and PC1 of the traits correlated for both male (r = −0.44, p < 0.001) and female voices (r = −0.70, p < 0.001). None of the other correlations turned out to be significant].

#### Influence of language on personality evaluations from voices

To further explore the influence of foreign language on personality impressions, we conducted a joint analysis considering data from native Scottish listeners (original experiment McAleer *et al*., 2014) and data from foreign Scottish listeners (Experiment 2). To do so, we included data from the two experiments in a single analysis. The hypothesis is that if ratings are similar in both experiments, similar PCA loadings should be obtained when considering native and foreign Hello ratings. The hypothesis was supported for both male and female voices, as the results revealed that valence-related traits mainly explained PC1 (PC1 male voices: Competence native: r = 0.86, p < 0.001; Trustworthiness foreign: r = 0.86, p < 0.001; PC1 female voices: Likeability native: r = 0.96, p < 0.001; Trustworthiness foreign: r = 0.88, p < 0.001) and Dominance/Aggressiveness mainly explained PC2 (PC2 male voices: Dominance native = r = 0.83, p < 0.001; Aggressiveness foreign: r = 0.77, p < 0.001; PC2 female voices: Aggressiveness native = r = 0.75, p < 0.001; Aggressiveness foreign: r = 0.79, p < 0.001).

Additionally, the relationship between the acoustic measures of voices and the joint PCAs was explored by means of a stepwise regression anlysis. For male voices, 53% of the variance of PC1 (R = 0.75, F (2, 29) = 19.1, p < 0.001) was explained by a linear combination of HNR (b = −0.72, p < 0.001, *VIF = *1.01) and alpha (b = 0.29, p = 0.02, *VIF = *1.01), while for female voices, 57% of the variance of PC1 (R = 0.78, F (3, 28) = 15.19, p < 0.001) was explained by a combination of dispersion (b = 0.70, p < 0.001, *VIF = *1.03), intonation (b = 0.44, p = 0.002, *VIF = *1.26) and glide (b = 0.31, p = 0.02, *VIF = *1.26). For PC2 of male voices, a combination of F0 (b = −0.64, p < 0.001, *VIF = *1.22), alpha (b = −0.55, p < 0.001, *VIF = *1.11) and glide (b = 0.28, p = 0.01, *VIF = *1.28), explained 68% of the variance (R = 0.84, F (3, 28) = 23.6, p < 0.001); for female voices, a combination of shimmer (b = 0.75, p = 0.02, *VIF = *1.67) and jitter (b = −0.41, p = 0.02, *VIF = *1.67) explained 30% of PC2 variance (R = 0.59, F (2, 29) = 7.9, p = 0.002).

### Discussion

In Experiment 2, we asked Spanish listeners to evaluate personality traits in their foreign language (English). We replicated the original study^3^ in several aspects. First, we found high inter-rater reliability for all the traits. Second, we obtained a two-dimensional social voice space, explaining most of the variance. For the first component (PC1), trustworthiness and competence for males and attractiveness, likeability, and trustworthiness for females were the most important traits. For the second component (PC2), aggressiveness and dominance were the most relevant traits for both female and male voices. These results suggest that first impressions from voices are not influenced by the language of the speaker, whether native or foreign. Also, these results suggest that the gender of the listener does not play a role in first impression evaluations. Female and male raters judged personality very similarly from voices as indicated by the nearly one-to-one correlations. While the two experiments support a two-dimensional social voice space, additional elements compose such space for evaluations in a foreign language. In particular, attractiveness (PC1) and aggressiveness (PC2) were important when creating the social voice space for foreign listeners but not for native listeners of English. Interestingly, all Spanish listeners, regardless of whether evaluations were made in their native or foreign language, associated attractiveness to valence and aggressiveness to strength/dominance. This observation supports the idea of culture influencing the weight of some personality traits in social evaluation.

Vocal measures had a similar influence on personality evaluations as in McAleer’s *et al*.^3^, except for pitch. For native and foreign listeners of English, fluctuations in HNR are the most robust acoustic cue when perceiving valence-related traits from male voices (trustworthiness, likeability): higher HNR was perceived as more trustworthy than lower HNR. This result is in line with previous works linking HNR to friendliness^14^. For female voices, changes in intonation and loudness/amplitude of the voices (i.e., shimmer) guided evaluations of trust and dominance. Notewithstanding, the relationship between pitch and dominance in the joint analysis and previous reports^3,5,13,15^ contrasts with the data obtained for the group of foreign listeners (male voices with a lower pitch were perceived as less dominant than high pitch ones). We do not have a clear explanation for this difference, but the fact that PC solutions were flipped with respect to McAleer *et al*.’s results might have influenced the results. Another possibility is that the relation between pitch and dominance is cross-linguistically modulated. As can be observed in Fig. 2 (lower panel), the correlation between PC2 (taken as an index of dominance) and pitch is negative for native Scottish listeners, but positive for both Spanish groups (although not significant for Spanish voices).

In sum, the results from Experiment 2 revealed that the language of the speaker, native or foreign, does not modulate personality impressions of listeners. Listeners are driven by the acoustical properties of the spoken language when evaluating the personality of the speaker, although the culture/language background of the listener also plays a role.

## General Discussion

In this article we explored two issues: a) how cross-linguistically generalizable the social voice space of perceived personality is^3^ and b) whether listeners can extract the same voice space when listening to foreign language speakers. To answer these questions, we tested Spanish listeners evaluating Spanish voices (Experiment 1) and Scottish voices (Experiment 2).

The results from the two experiments revealed that listeners form personality impressions from voices very rapidly, even from brief utterances containing limited information (one single word; Hello/Hola;^3,37,38^). Ratings of perceived personality were highly consistent among listeners regardless of the language in which voices were evaluated. Furthermore, a two-dimensional social voice space was obtained in both experiments, with traits such as trust, confidence, likeability, and warmth having an important weight in explaining variance of the first principal component (PC1) and traits such as dominance, aggressiveness, and competence explaining most of the variance of PC2. Additionally, Spanish listeners rated Scottish voices similarly to native Scottish listeners, with valence (as a summary of trustworthiness, likeability, warmth) and dominance (as a summary of aggressiveness, competence) as two of the main personality traits driving first-impressions. That is, Spanish listeners equally evaluated speakers of their native and foreign language for valence and dominance. These results replicate previous evidence showing the rapid attribution of personality traits to novel voices^4^ and novel faces^40^, with personality traits related to valence and dominance accounting for the majority of the data.

The two-dimensional social space, summarized by valence and dominance^2,3,43^ supports the idea that evaluation of these two personality traits is evolutionary relevant. This suggests that vocal expressions might possess some invariant properties that allow listeners to form fast impressions independently of their gender, culture or linguistic ability. Obtaining information about the intent of others helps individuals to appropriately evaluate whether to approach or to avoid interaction with them^3,6^. Listeners are consistent in their judgements of first impressions from voices across gender^3^, language^2,43^, and whether it is a native or foreign language to them. There are, however, subtle differences across languages in the way the social voice space is created. For instance, Scottish listeners rated attractiveness from male voices as related to dominance, while for Spanish listeners the same trait was related to valence. That is, attractive male voices were perceived as dominant for Scottish listeners, while for Spanish listeners the same voices were perceived as trustworthy. Interestingly, Spanish listeners judge attractiveness similarly in their native (Experiment 1) and foreign language (Experiment 1), which suggests that the influence of personality traits on first impressions might be culturally mediated for some traits but not for others. These results support the idea that the social voice space is used in every language but culture accentuates some dimensions over others^1^.

Our findings, however, contrast with the idea that speakers of a foreign language are evaluated as less trustworthy, intelligent or competent than speakers sharing the language of the listener^30,44^. There might be several reasons for this difference, one of those being the amount of information provided to the listener. While here we presented one isolated word, previous studies presented listeners with an entire passage from a speaker. The cognitive burden of processing a foreign language may have resulted in a “halo” effect (cognitive bias in which an observer’s impression of a person influences the evaluation of their traits^45^) for the listener. That is, because I do not understand you well, I consider you less trustworthy. It is possible that by presenting only one-word, listeners do not experience such processing difficulty, allowing unbiased personality impressions to be formed. Note that while this is an interesting possibility, it would contrast with those studies showing that listener’s first impressions (in their native language) do not seem to be modulated by the quantity of information provided (one word/entire paragraph^37,46^) or the content of such information (normal vs. reversed speech^47^). A second possibility relates to language familiarity. That is, because our participants were proficient in English, they were necessarily familiar with the phonetic properties of the word “Hello”, which might have reduced the influence of foreign language in personality impressions. The influence of familiarity has been explored in other domains, such as face evaluation^40^ showing that familiarity affects the reliability of the ratings among perceivers; familiarity with the race increases agreement between raters. Different studies^47,48^ have addressed the issue of whether language comprehension or language familiarity is needed for voice perception. It would be interesting to test in future studies the influence of familiarity on personality evaluation. It would probably require changing the language from English to another less widely known language (or rather testing populations that are not familiar with the word Hello).

Our results have also revealed that while perceived personality traits from voices generalize across languages, the vocal properties of speech that listeners pay attention to do not. In McAleer *et al*.’s study^3^, trustworthiness was mainly related to variations in pitch (F0) for male voices and to intonation and glide (how the pitch moves) for female voices while dominance was related to pitch for male and female voices. For both genders high-pitch voices were judged as more trustworthy, whereas high-pitch voices were judged as more dominant for female voices and less dominant for male voices. Conversely, our results revealed that none of the vocal properties taken from McAleer *et al*.^3^, played a role when judging trust or dominance from Spanish voices. It is possible that these cross-language differences regarding the relationship between acoustic measures and personality traits arise from a different prototype of what a trustworthy or dominant voice is for the Spanish culture relative to and other cultures. Scherer^1^ attributed cross-cultural differences in the attribution of personality traits to speakers, wittingly or unwittingly, manipulating their expressive cues to project a socially desirable trait, making, in turn, those cues more preponderant for listeners. In his study, German and American listeners identified sociability of American speakers and dominance of German speakers, two stereotype traits of the American and German society respectively (see also^25^, for cross-cultural similarities and differences during personality evaluation).

Regarding dominance, our results are more difficult to reconcile with previous research^14,16^. Pitch has been previously shown to be negatively related to androgen levels (testosterone), which provides signals of genetic quality (reproductive success) and gauges competence and dominance both in humans^5,13,37,49^ and in non-human animals^50^. Thus, pitch seems to be a reliable cue of physical dominance (but see^51,52^), which makes one wonder why no effect was observed in Spanish. Spanish and Scottish voices did not differ in their average F0. Therefore differences cannot be attributed to how pitch is distributed across languages. Remarkably, although direct correlations between PC2 and pitch were not statistically significant for Spanish voices, the direction of the correlation was positive for both groups of Spanish listeners while negative for Scottish participants (Fig. 4). That is, low-pitch voices were judged as less dominant than high-pitch voices for Spanish participants. In speculating about the opposite effect of pitch on dominace between languages, differences in the traits loading PC2 might be at the origin of such discrepancy. In particular, aggressiveness was the main trait driving PC2 for Spanish voices (while dominance for Scottish voices), which might influence the way pitch is perceived by listeners. As is has been suggested, the influence of pitch on social impressions may vary depending on the context. For instance, Klofstad^53^ showed that in the 2012 US election, male candidates with higher-pitched voices were more successful when running against female opponents. This result was interpreted as male candidates with lower voices being perceived as too aggressive when paired to a female opponent. At any rate, our results suggest that the relationship between pitch and dimensions related to strength^33,54,55^, competence^12^ or dominance^56^ for male voices, are modulated by the linguistic/cultural background of the listener.

## Conclusion

In sum, in two experiments, we have provided evidence that first-impressions from voices are extracted very rapidly and appear to be generalizable across languages. However, the vocal acoustics listeners pay attention to when evaluating personality from voices seem to be culturally/linguistically determined.

## Electronic supplementary material

Supplementary Material
