# Supplementary material for: Forming social impressions from voices in native and foreign languages
Source: Sci Rep. 2019 Jan 23;9:414. doi: 10.1038/s41598-018-36518-6 (PMC6344506; doi:10.1038/s41598-018-36518-6)
Supplement: Supplementary file 1 — Supplementary Material [file 41598_2018_36518_MOESM1_ESM.doc]

**Forming social impressions from voices in native and foreign languages**

Cristina Baus, Phil McAleer, Katherine Marcoux, Pascal Belin & Albert Costa

**SUPPLEMENTARY MATERIAL**


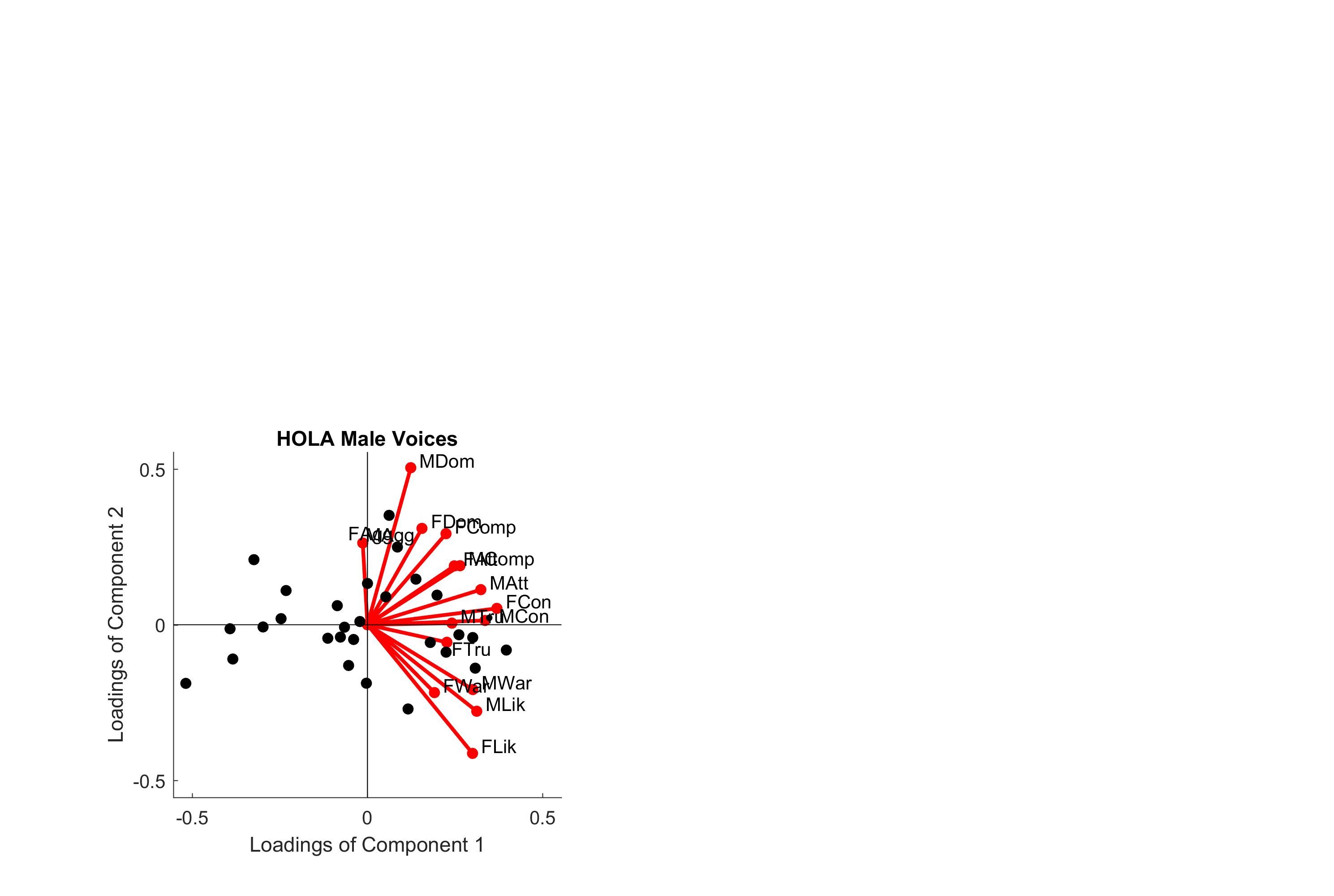

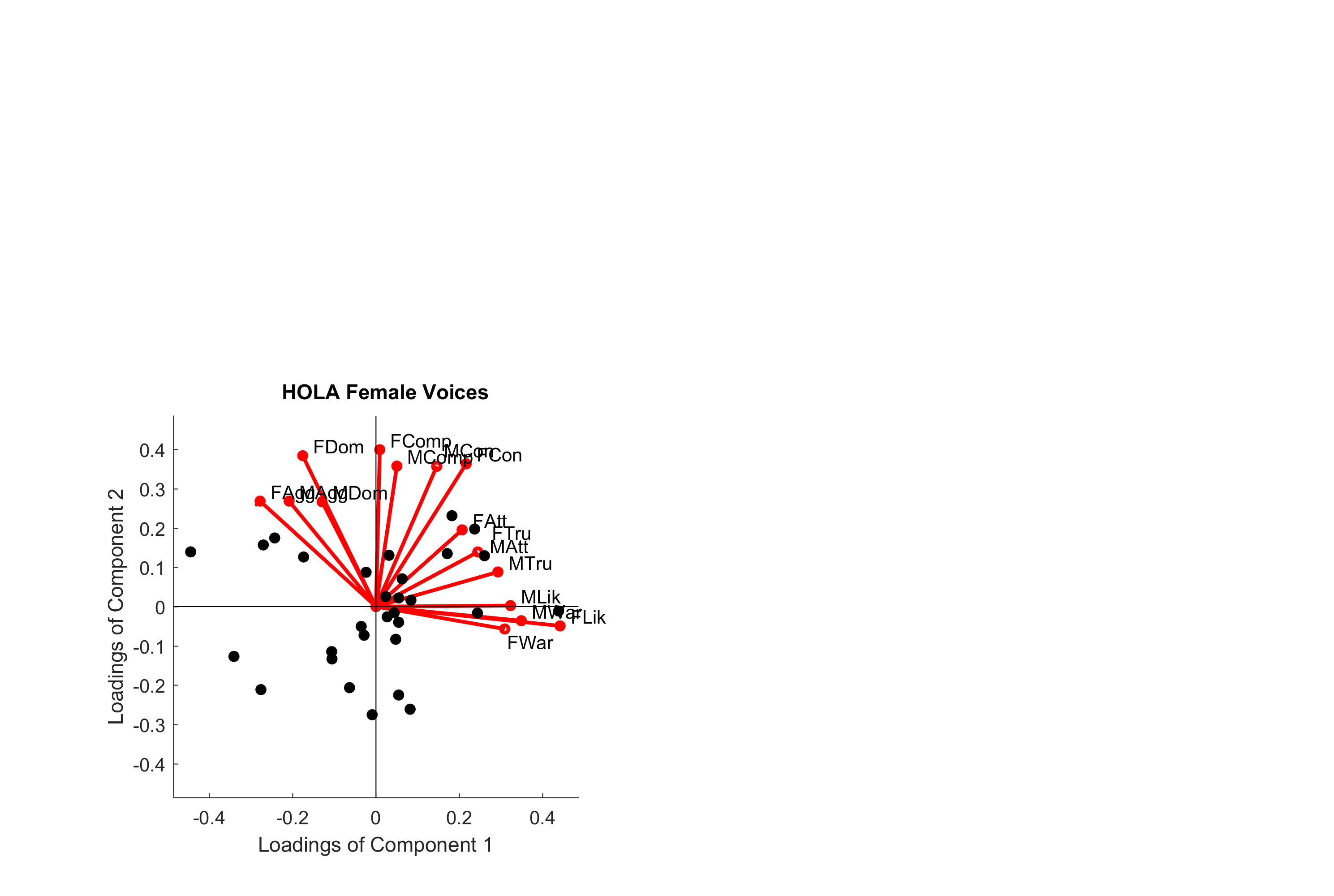


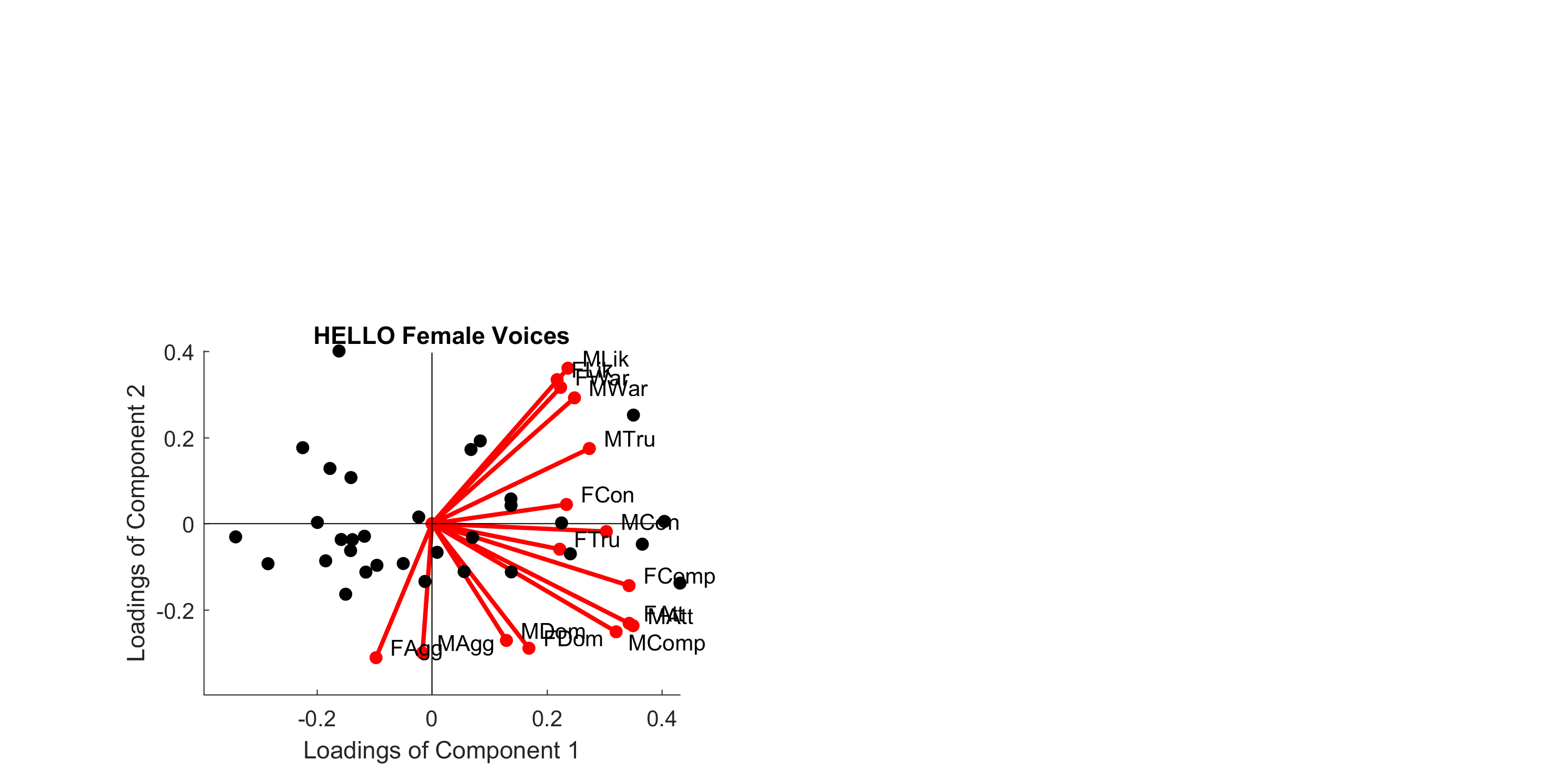

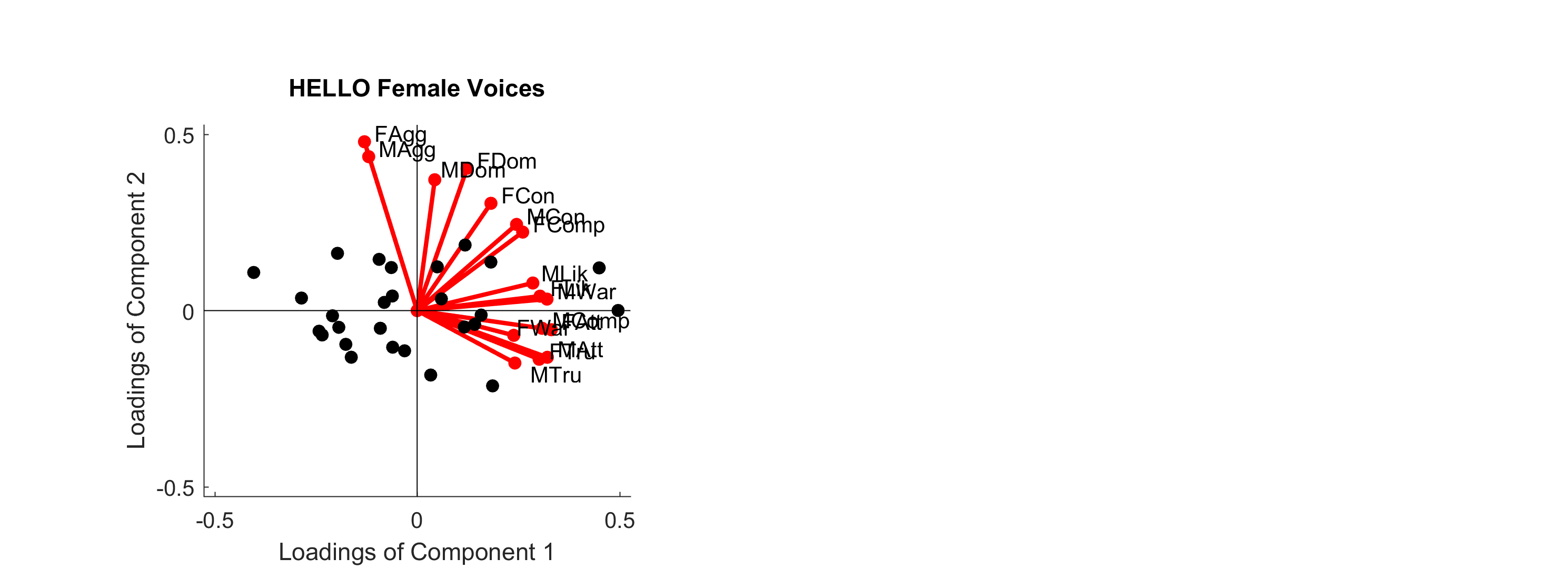


|  | **HOLA** | | | | **HELLO** | | | |
| --- | --- | --- | --- | --- | --- | --- | --- | --- |
|  | Female voices | | Male voices | | Female voices | | Male voices | |
| Trait | **PC1** | **PC2** | **PC1** | **PC2** | **PC1** | **PC2** | **PC1** | **PC2** |
| M_Agg | -0,64 | 0,60 | -0,06 | 0,65 | -0,43 | 0,81 | -0,07 | -0,78 |
| M_Att | 0,74 | 0,30 | 0,92 | 0,18 | 0,92 | -0,19 | 0,88 | -0,37 |
| M_Com | 0,17 | 0,88 | 0,79 | 0,32 | 0,88 | -0,07 | 0,84 | -0,41 |
| M_Con | 0,41 | 0,74 | 0,93 | 0,02 | 0,78 | 0,40 | 0,87 | -0,03 |
| M_Dom | -0,50 | 0,75 | 0,38 | 0,87 | 0,19 | 0,81 | 0,53 | -0,69 |
| M_Lik | 0,93 | 0,01 | 0,85 | -0,43 | 0,90 | 0,13 | 0,69 | 0,65 |
| M_Tru | 0,89 | 0,20 | 0,92 | 0,01 | 0,75 | -0,24 | 0,87 | 0,34 |
| M_War | 0,95 | -0,07 | 0,86 | -0,34 | 0,91 | 0,05 | 0,69 | 0,51 |
| F_Agg | -0,78 | 0,55 | -0,06 | 0,65 | -0,45 | 0,84 | -0,40 | -0,79 |
| F_Att | 0,64 | 0,45 | 0,78 | 0,34 | 0,92 | -0,08 | 0,87 | -0,37 |
| F_Com | 0,03 | 0,87 | 0,75 | 0,55 | 0,87 | 0,38 | 0,93 | -0,24 |
| F_Con | 0,57 | 0,70 | 0,94 | 0,08 | 0,63 | 0,53 | 0,86 | 0,10 |
| F_Dom | -0,50 | 0,81 | 0,62 | 0,70 | 0,47 | 0,79 | 0,64 | -0,68 |
| F_Lik | 0,96 | -0,08 | 0,77 | -0,60 | 0,90 | 0,06 | 0,68 | 0,65 |
| F_Tru | 0,85 | 0,36 | 0,86 | -0,12 | 0,95 | -0,22 | 0,81 | -0,13 |
| F_War | 0,95 | -0,13 | 0,79 | -0,51 | 0,83 | -0,12 | 0,72 | 0,63 |
|  |  |  |  |  |  |  |  |  |

Figure XX. Principal Component Analysis solutions and main correlates of the Social Voice Space for “Hola” voices considering the gender of the participant. The two-dimensional solution of the PCA for male (left) and female (right) voices (black dots). Labels equate to: Agg – Aggressiveness; Att-Attractiveness; Comp- Competence; Con – Confidence; Dom – Dominance; Lik- Likeability; Tru – Trustworthiness; War – Warmth. Labels starting with M stand for male participants and beginning with F for female participants.

Experiment 1: PCAs considering the gender of the participants

Male voices PCA. A two-dimensional soluction accounted for 83% of the variance. PC1 explained 63% of the variance and PC2 the 20%. The personality traits that most strongly loaded for PC1 were confidence and trustworthiness, both from females’ evaluation (confidence, r =0.93, p < .001; trustworthiness r =0.85, p < .001) and males’s evaluations (r =0.93, p < .001 and r =0.93, p < .001) and additionally attractiveness from males’ evaluation (r =0.92, p < .001 and r =0.77, p < .001 for males and females respectively). For PC2, dominance was the trait that loaded the most (r =0.87, p < .001 and r =0.70, p < .001, for females and males respectively).

Female voices PCA. A two-dimensional solution accounted for 82% of the variance (PC1: 53%, PC2: 29%). As for the general analysis, the two personality traits that most strongly loaded PC1 were likeability (r =0.96, p < .001 and r =0.92, p < .001 for females and males respectively) and warmth (r =0.95, p < .001 and r =0.95, p < .001 for females and males respectively), both from females and males’ evaluations. For PC2, competence (r =0.87, p < .001 and r =0.88, p < .001 for females and males respectively) and dominance (r =0.80, p < .001 and r =0.75, p < .001 for females and males respectively) loaded the most.

Loadings of the two components from female and male participants were correlated to further explore the influence of participant’s gender on our results. Correlations were very high both for male (*r* =0.97, p <0.001 for PC1 loadings and *r* =-0.93, p <0.001 for PC2) and female voices (*r* =0.95, p <0.001 for PC1 loadings and *r* =0.89, p <0.001 for PC2). Altogether, these results suggest that the evaluation of personality traits does not seem to be influenced by the gender of the listener.

Experiment 2: PCAs considering the gender of participants

Male voices PCA. A two-dimensional soluction accounted for 83% of the variance. PC1 explained 60% of the variance and PC2 the 23%. The personality traits that most strongly loaded for PC1 were competence from females’ evaluation (r =0.92, p < .001, r=0.83, p<0.001 for females and males respectively) and attractiveness from males’ evaluations (r =0.88, p < .001, r =0.87, p < .001, for males and females respectively). For PC2, aggressiveness was the trait that loaded the most both from females’ and males’ evaluations (r =0.78, p < .001 and r =0.78, p < .001 respectively).

Female voices PCA. A two-dimensional soluction accounted for 81% of the variance (PC1: 64%, PC2: 16%). The personality traits that most strongly loaded for PC1 were attractiveness from females’ and males’ evaluations (r =0.92, p < .001 and r =0.91, p < .001 respectively), likeability from females’ evaluation (r =0.90, p < .001) and trustworthiness and warmth from males’ evaluation (r =0.91, p < .001, r =0.90, p < .001). For PC2, aggressiveness (r =0.84, p < .001 and r =0.81, p < .001 for females and males respectively) loaded the most.

Male and female loadings of the two components were correlated to further explore the influence of participant’s gender on personality impressions. Correlations were very high both for male (*r* =0.95, p <0.001 for PC1 loadings and *r* =0.94, p <0.001 for PC2) and female voices (*r* =0.96, p <0.001 for PC1 loadings and *r* =0.90 p <0.001 for PC2).
